# Quality comparison of a state-of-the-art preparation of a recombinant L-asparaginase derived from *Escherichia coli* with an alternative asparaginase product

**DOI:** 10.1371/journal.pone.0285948

**Published:** 2023-06-15

**Authors:** Arndt Schnuchel, Christoph Radcke, Lars Theobald, Stefan Doeding

**Affiliations:** 1 medac GmbH, Wedel, Germany; 2 Celares GmbH, Berlin, Germany; 3 Nordmark Pharma GmbH, Uetersen, Germany; University of Nebraska Medical Center, UNITED STATES

## Abstract

L-asparaginase (ASNase) is a protein that is essential for the treatment of acute lymphoblastic leukemia (ALL). The main types of ASNase that are clinically used involve native and pegylated *Escherichia coli* (*E*. *coli*)-derived ASNase as well as *Erwinia chrysanthemi-*derived ASNase. Additionally, a new recombinant *E*. *coli*-derived ASNase formulation has received EMA market approval in 2016. In recent years, pegylated ASNase has been preferentially used in high-income countries, which decreased the demand for non-pegylated ASNase. Nevertheless, due to the high cost of pegylated ASNase, non-pegylated ASNase is still widely used in ALL treatment in low- and middle-income countries. As a consequence, the production of ASNase products from low- and middle-income countries increased in order to satisfy the demand worldwide. However, concerns over the quality and efficacy of these products were raised due to less stringent regulatory requirements. In the present study, we compared a recombinant *E*. *coli*-derived ASNase marketed in Europe (Spectrila^®^) with an *E*. *coli*-derived ASNase preparation from India (Onconase) marketed in Eastern European countries. To assess the quality attributes of both ASNases, an in-depth characterization was conducted. Enzymatic activity testing revealed a nominal enzymatic activity of almost 100% for Spectrila^®^, whereas the enzymatic activity for Onconase was only 70%. Spectrila^®^ also showed excellent purity as analyzed by reversed-phase high-pressure liquid chromatography, size exclusion chromatography and capillary zone electrophoresis. Furthermore, levels of process-related impurities were very low for Spectrila^®^. In comparison, the *E*. *coli* DNA content in the Onconase samples was almost 12-fold higher and the content of host cell protein was more than 300-fold higher in the Onconase samples. Our results reveal that Spectrila^®^ met all of the testing parameters, stood out for its excellent quality and, thus, represents a safe treatment option in ALL. These findings are particularly important for low- and middle-income countries, where access to ASNase formulations is limited.

## Introduction

L-asparaginase (ASNase) is a high molecular weight enzyme of approximately 140 kDa consisting of four identical subunits. ASNase is active as a homotetramer [1, 2]. The enzyme breaks down plasma L-asparagine to L-aspartic acid and ammonia, which results in rapid and complete depletion of asparagine. For leukemic cells, asparagine is an essential amino acid, and therefore an asparagine-depleted environment induces cell death, while normal cells have the capacity to synthesize this amino acid themselves [3, 4]. In clinical use, ASNase is indicated as a component of an antineoplastic combination therapy for the treatment of acute lymphoblastic leukemia (ALL) in pediatric patients from birth to 18 years as well as adults and has been tested in several clinical studies [5].

Spectrila^®^ (medac), the first recombinant *E*. *coli*-derived asparaginase preparation has received marketing approval by the European Medicines Agency (EMA) in 2016 [6].

In European countries and the Unites States, strict requirements for quality as defined by the regulatory authorities, e.g., EMA or U.S. Food and Drug Administration (FDA), must be met by all products [7–9]. Controlled trials against established ASNase preparations are also required to receive marketing authorization for biosimilar products, thus guaranteeing safety and efficacy for the patient.

New generic *E*. *coli*-derived ASNase preparations have been approved for the treatment of ALL in several non-European countries [10]. Yet, these products may find their way into other markets, in part as a less costly alternative to existing ASNases and also to reduce the risk of shortage of ASNases. However, in comparison to products with EMA or FDA market authorization there are less stringent regulatory conditions for these imported products, e.g., only based on registration in the country of origin and Good Manufacturing Practices certificate [11]. Furthermore, data from clinical studies are commonly not available or even non-existent, and data on the analytical comparability are rare and difficult to generate partly due to limited material availability. One of these new ASNase products approved in non-European countries is Onconase, an ASNase product manufactured in India. It is not publicly known whether the product has been tested in a clinical trial. The use of such preparations may cause serious side effects like increased rate of allergic reactions thereby leading to a worse patient outcome [4, 10, 11].

In this thorough head-to-head quality investigation, Onconase was analyzed in comparison to Spectrila^®^ as state-of the-art centrally registered medicinal product in Europe and several non-European countries. The two products were compared based on a set of typical testing methods for pharmaceutical protein products.

## Materials and methods

### ASNase preparations

The tested ASNases were obtained as lyophilizates in vials (Table 1). To minimize a potential impact of sample preparation on the analytical methods, the identical dilution buffers, dilution scheme and sample handling were applied in parallel for Onconase (lot number: LALH9B3) and Spectrila^®^. For analysis, the samples were dissolved in water for injection, and further diluted and aliquoted as required for the specific test methods. Storage could potentially impact the results of some methods. Therefore, appearance, visible particles, protein concentration, purity by reversed-phase high-pressure liquid chromatography (RP-HPLC) and size exclusion chromatography (SEC) were directly measured with diluted samples, while test aliquots for other methods were frozen until usage. A reference substance for Spectrila^®^ was included for some assays according to the analytical protocol.

**Table 1:** Tested ASNase preparations.

Trade name	Company	Declared potency
Spectrila^®^	medac, Germany	10,000 U/vial
Onconase	United Biotech Pvt Ltd, India	10,000 U/vial

### Appearance and quantity

For quality comparison, standard methods for pharmaceutical protein products were applied.

### Color

The clarity, degree of opalescence and degree of coloration were visually examined based on Ph. Eur. 2.2.1. and 2.2.2.

### Visible particles

The test for visible particles was executed according to Ph. Eur. 2.9.20.

### Protein concentration (UV)

The UV absorbance (at 278 nm) was measured based on standard procedures; spectrophotometry and total protein determination were performed according to Ph. Eur 2.2.25 and 2.5.33 (method 1).

### Identity

#### Tryptic peptide mapping

The test items (150 μg each) were subjected to standard protocol [12] for tryptic digestion in solution without prior reduction and alkylation.

Analysis of peptides was performed by using high-performance liquid chromatography-tandem mass spectrometry (HPLC-MS/MS) with an HPLC system coupled with an Orbitrap Velos MS. Sequence coverage and sequence identity were analyzed by corresponding database search and software.

#### Molecular weight determination

For the determination of molecular weight as identity parameter and for purity, sodium dodecyl-sulfate polyacrylamide gel electrophoresis **(**SDS-PAGE) was performed under reducing and non-reducing conditions by using SDS-PAGE gels. Prior to SDS-PAGE, samples were heated up. The gels were stained with SimplyBlue™ SafeStain kit (Invitrogen, USA) according to the manufacturer’s instructions.

### Potency

#### Enzymatic activity

For analysis of ASNase activity, a commercially available kit (Sigma-Aldrich, Germany) [13] was used according to the manufacturer`s instruction. Standard methods for pharmaceutical protein products were applied.

#### Protein purity

Purity of the tested ASNases was determined by using SDS-PAGE as described above, RP-HPLC and SEC. The chromatographic system for RP-HPLC consisted of a standard RP column (YMC pack Protein RP column 4.6 x 250 mm) and Waters Alliance HPLC unit. Solvents used were acetonitrile,water and trifluoroacetic acid. Detection was carried out at a wavelength of 220 nm. The chromatographic system for SEC consisted of a standard size exclusion column (TSK-Gel column G2000SWXL 7.8 x 300 mm) and Waters Alliance HPLC unit and was performed according to instructions of the manufacturer. Detection was carried out at a wavelength of 220 nm.

#### Analysis of isoforms

Capillary zone electrophoresis (CZE) was performed to identify possible isoforms of the ASNases. CZE was performed according to the analytical protocol [14].

### Host cell contaminations

#### Analysis of host cell DNA

Residual *E*. *coli* host cell DNA as process-related impurity was determined by using a commercially available assay (Picogreen assay, Invitrogen, USA). Analysis was performed according to the manufacturer’s protocol.

#### Analysis of host cell protein (HCP)

For *E*. *coli* host cell proteins as process-related impurity, a specific in-house sandwich enzyme linked immunosorbent assay (ELISA) assay was developed against the production strain for Spectrila^®^ (not commercially available, BioGenes, Germany). The detection of HCPs was evaluated by using specific polyclonal antibodies. The ELISA was performed according to the manufacturer´s instructions.

### Safety

#### Analysis of endotoxins

An LAL assay was performed using a commercially available kit (Endosafe, Charles River, UK) according to the manufacturer’s instructions.

## Results

### Summary of analytical testing

Quality testing of Spectrila^®^ and Onconase included analysis for different analytical parameters, such as identity, potency and purity. Major differences were found for Spectrila^®^ and Onconase. For all examined criteria, Spectrila^®^ met the quality targets/target ranges, indicating a highly pure and effective ASNase. Onconase, however, failed the quality targets/target ranges for almost all criteria (Table 2).

**Table 2:** Comparison of Spectrila^®^ and Onconase for relevant quality attributes.

Analytical parameter and method		Target / Target Range	Spectrila^®^	Onconase
**Appearance and description**				
Color		Clear	Clear	Clear
Visible particles		None	None	Present at dilution*
**Protein quantification**				
Protein concentration		40–48 mg/vial	40 mg/vial	40 mg/vial
**Identity**				
Molecular weight (SDS-PAGE)		34–38 kDa	36 kDa	36 kDa
**Potency**				
Enzymatic activity		80–120%	97%	70%*
**Purity**				
SDS-PAGE		≥ 95%	100%	75%*
RP-HPLC		≥ 98%	99%	86%*
SEC	Aggregates	≤ 3.0%	0.3%	13.5%*
	Tetramer	≥ 95%	99.4%	82.7%*
	Monomer	≤ 2.0%	0.3%	3.8%*
**Heterogeneity**				
CZE	Main isoform	≥ 84%	88%	41%*
	Acidic isoforms	≤ 8%	6%	26%*
	Basic isoforms	≤ 8%	6%	33%*
**Host cell contaminations**				
DNA		N/A	868 pg/mg	10,162 pg/mg
HCP (ELISA)		≤ 100 ppm	1 ppm	> 500 ppm*
**Safety**				
Endotoxins (LAL assay)		≤ 10 IU/vial	< 2 IU/vial	26 IU/vial*

* Failed target / target range

### Two variants of primary structure found for Onconase

**Fig 1:**
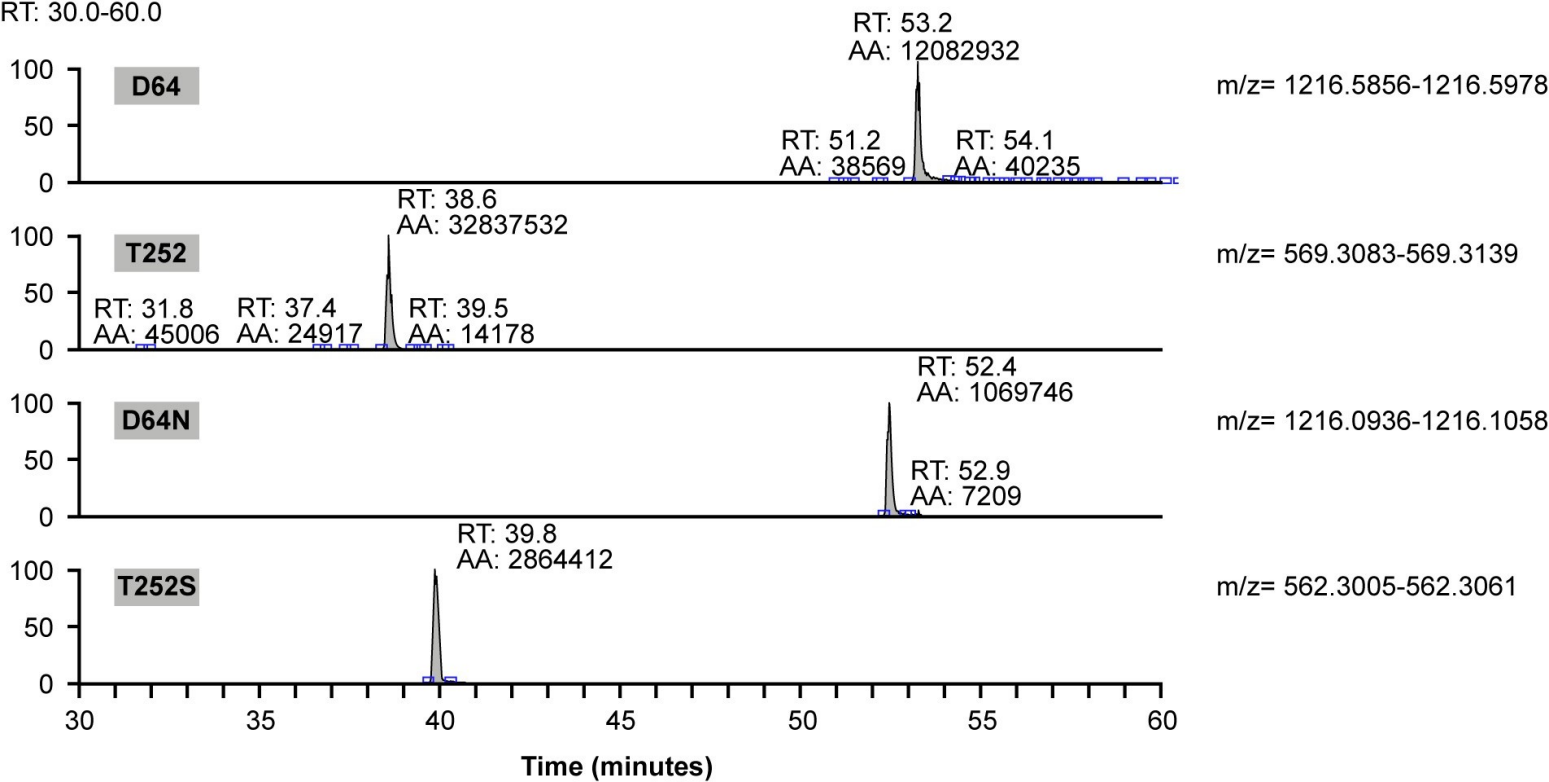
Extracted ion chromatograms of peptides with amino acid changes. Two amino acid changes were detected in the Onconase sample, while no amino acid change was observed for Spectrila^®^. Analysis of the peak areas indicates that 10% of D64N and T252S occurred in the Onconase sample. The accession number for asparaginase is: Uniprot P00805. Both asparaginases analyzed did not bear a tag neither at the N- or C-terminus, respectively.

The tryptic peptides were measured with HPLC-MS/MS and evaluated for accurate mass. The sequence coverage was 98.8% for both test samples. However, in the Onconase sample, a partial exchange of two amino acids could be detected: in position 64 from aspartic acid (D) to asparagine (N) and in position 252 threonine (T) to serine (S). Analysis of the area of the peaks in extracted ion chromatograms of these two peptides indicates that 10% of these amino acids are changed in Onconase (Fig 1, Table 3). No amino acid change was observed in Spectrila^®^.

**Table 3:** Identification of amino acid variants in Onconase.

Amino acid change	Spectrila^®^	Onconase
**Peptide 49–71 and variant**		
GEQVVNIGSQDMNDDVWLTLA	100%	90%
GEQVVNIGSQDMND **N** VWLTLA	Not detected	10%
**Peptide 252–261 and variant**		
TVFDTLATAAK	100%	90%
**S** VFDTLATAAK	Not detected	10%

### Onconase showed reduced potency and purity

The potency was measured as enzymatic activity. Spectrila^®^ exhibited an enzymatic activity that corresponds to 97% of the nominal activity of 10,000 Units/vial. In contrast, Onconase exhibited only 70% of the nominal enzymatic activity (Table 2).

**Fig 2:**
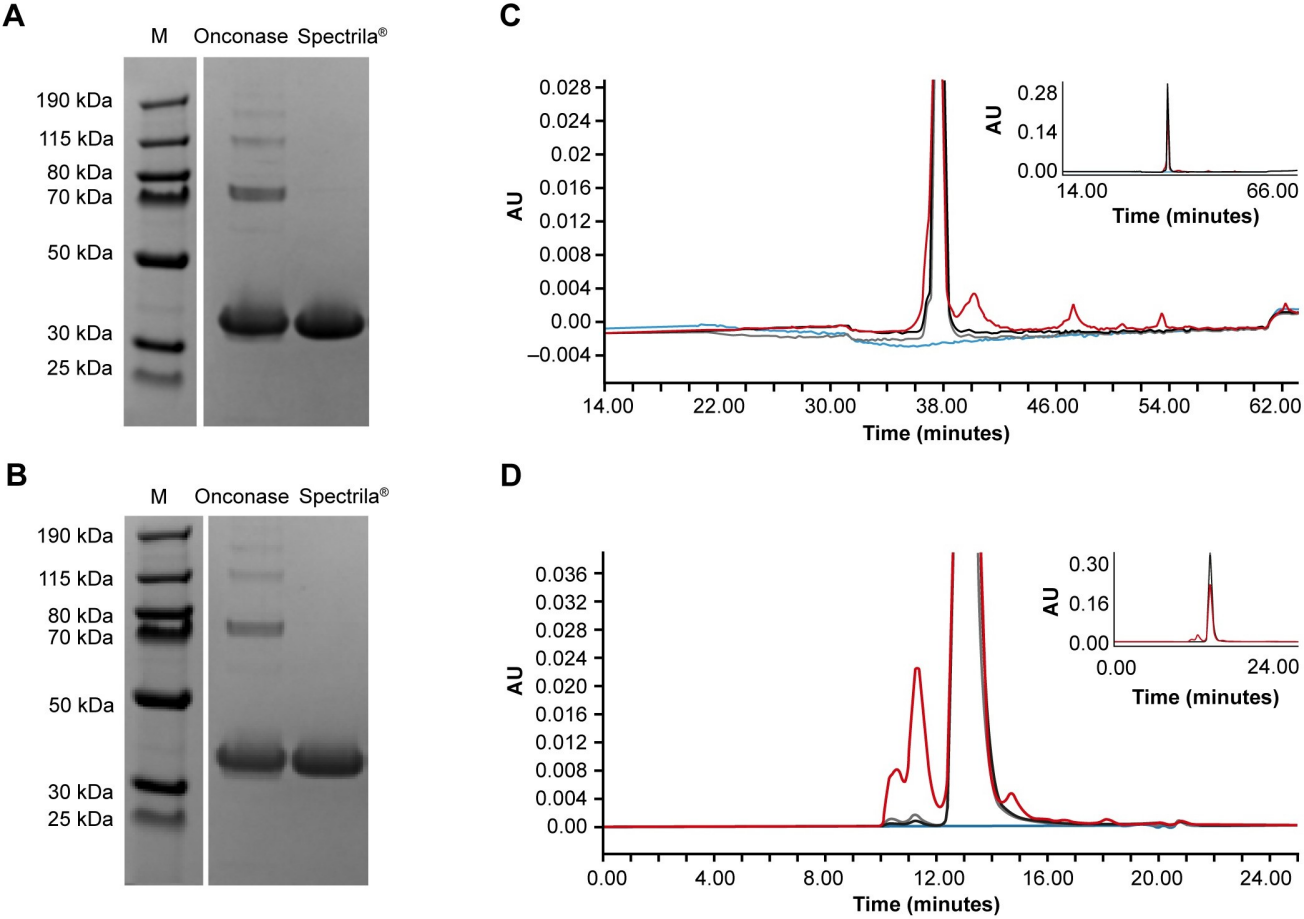
Analysis of protein purity. (A) Onconase and Spectrila^®^ showed distinct bands for the ASNase monomer at 36 kDa as shown by SDS-PAGE under non-reducing conditions. However, multiple additional bands at higher molecular weight were present in the Onconase sample only. (B) The same result was obtained under reducing conditions. The Onconase sample showed several additional bands. (C) As analyzed by RP-HPLC, Spectrila^®^ (black) shows a single peak without any additional side peaks, which is congruent with the peak of the reference standard (grey). Onconase (red) shows a broader main peak and several additional peaks at higher retention times. A blank sample is shown in blue. (D) SEC analysis revealed minimal aggregate content and a strong main peak for Spectrila^®^ (black). For Onconase (red) more pronounced peaks are found at lower retention times, and at higher retention times additional peaks were present compared to the reference standard (grey). The blank sample is shown in blue.

Molecular weight and purity were analyzed by SDS-PAGE under reducing and non-reducing conditions, respectively, to identify protein subunits or impurities linked through disulfide bridges. Due to the sample preparation and electrophoresis conditions, the ASNase tetramer is dissociated to monomers. Spectrila^®^ showed a single band in SDS-PAGE at the molecular weight of approximately 36 kDa demonstrating its high purity of nearly 100% (Table 2, Fig 2A and 2B). For Onconase, we found multiple bands at higher molecular weight at approximately 72 kDa for the ASNase dimer and at approximately 120 kDa and 173 kDa corresponding to impurities and higher order aggregates. Based on the quantitative evaluation of the SDS-PAGE, the purity of Onconase was only 75%. No differences between reducing and non-reducing conditions could be observed. The analysis of Spectrila^®^ by RP-HPLC revealed a single peak in line with its high purity (99% main peak) (Table 2, Fig 2C). In contrast, we found a broader main peak (86%) for Onconase and at least three additional peaks. We assume that these peaks correspond to undesired protein impurities not present in Spectrila^®^. In SEC, Spectrila^®^ showed a strong peak with minimal aggregate or monomer content (0.3% each) again indicative of its high purity (99.4% tetramer) (Table 2, Fig 2D). The lower purity of Onconase is also confirmed in the SEC profile with a pronounced aggregate content (13.5%) and, additionally, a distinct monomer peak (3.8%).

### Higher charge heterogeneity revealed for Onconase

**Fig 3:**
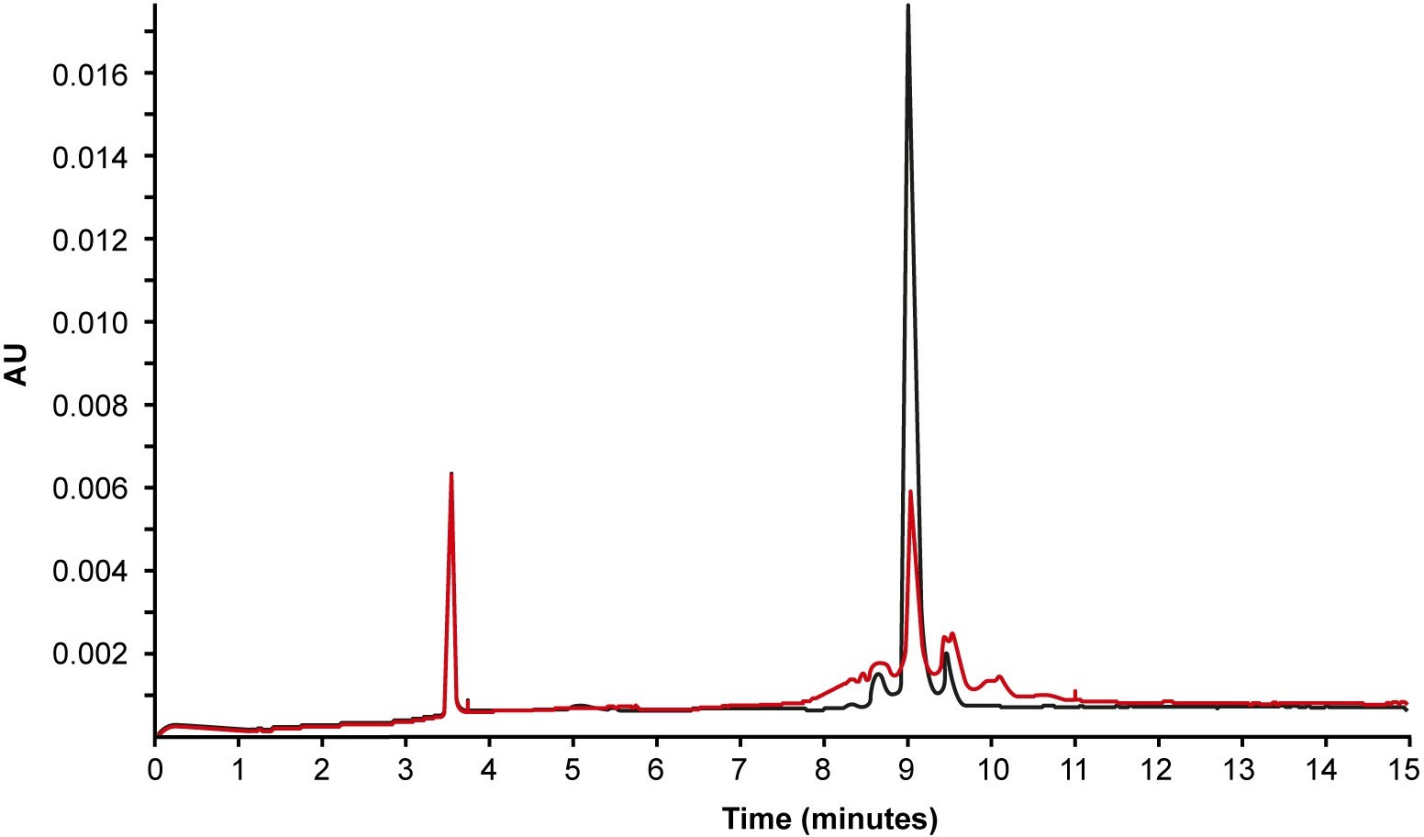
Identification of charge variants. The Onconase sample (red) showed a significantly reduced main peak with a strongly increased proportion of both acidic and basic isoforms by CZE analysis. The nature of these isoforms in Onconase is not known. The relative peak area of the main isoform for the Spectrila^®^ sample (black) was more than twice as large as the relative peak area of the main isoform for the Onconase sample.

The charge heterogeneity was evaluated by CZE. In the respective electropherogram, Spectrila^®^ showed a dominant main peak (88%). The content of acidic isoforms (two distinct peaks before main peak) and basic isoforms (one distinct peak after main peak) is comparable with 6% each (Table 2, Fig 3). In contrast, Onconase showed much higher content of acidic (26%) and basic (33%) isoforms, and consequently a significant decrease of the main peak area (41%) is observed compared to Spectrila^®^. In addition, two new peaks were present in the basic peak group, which could not be observed for Spectrila^®^.

### Onconase contained a high degree of process-related impurities

The measured DNA content of *E*. *coli* for Onconase (10,162 pg/mg) was almost 12-fold higher than for Spectrila^®^ with 868 pg/mg (Table 2). The content of *E*. *coli* HCP for Onconase determined with specific in-house ELISA is more than 300-fold higher than for Spectrila^®^. Even after 1:100 dilution of the Onconase sample, the concentration seemed much higher than the least diluted sample of the standard dilution series. Therefore, no valid quantitative result could be obtained. This finding is even more striking as the assay is specific for Spectrila^®^, and it is thus assumed that the actual HCP concentration is even higher than determined by the HCP assay applied here (Table 4). For Spectrila^®^, only 1 ppm of HCPs was measured. Additionally, the presence of endotoxins was much higher in the Onconase sample compared with Spectrila^®^ (26 IU/vial vs. < 2 IU/vial) and exceeded the target limit (Table 2). These results demonstrate the very high quality of Spectrila^®^ with regard to process-related impurities (Table 2, Table 4).

**Table 4:** Identification of HCP contamination.

Sample	Dilution	HCP concentration (ng/mL)	HCP/protein (ng/mg)	HCP mean (ppm)
Spectrila^®^	1:5	1.41	0.70	0.825
Spectrila^®^	1:10	1.90	0.95	
Onconase	1:50	> 250	> 125	> 250
Onconase	1:100	> 500	> 250	

Another general observation was the precipitation of Onconase upon dissolution. Clear solutions were expected for both ASNases after dissolving the lyophilizates. However, during further dilution, the appearance of visible particles was observed for Onconase. Interestingly, the precipitates seemed to not impact the concentration of ASNase. This phenomenon was not observed for Spectrila^®^.

## Discussion

The in-depth characterization of Spectrila^®^ revealed striking quality differences compared with Onconase. Spectrila^®^ met all the quality criteria examined, while Onconase showed clear deficits in terms of potency, purity and safety. In particular, purity analysis of Onconase by SDS-PAGE and SEC revealed the presence of impurities and higher order aggregates (i.e. 8-mer and 12-mer) not present in Spectrila^®^. The enzyme activity of Spectrila^®^ was close to 100% and well within the target range. The enzymatic activity of Onconase, however, was found to be only 70% of the label claim and below the lower target limit of 80%. Moreover, peptide mapping revealed the presence of at least two different asparaginase enzymes present in Onconase. These findings indicate that Spectrila^®^ can be used as a safe and effective treatment option in ALL. A recent study by Sidhu et al. investigated quality attributes of Spectrila^®^ and seven new ASNases in vitro and in vivo. The authors, similarly to our quality measurements, found excess of impurities and high levels of aggregates in almost all of the seven native *E*. *coli*-derived ASNases, while Spectrila^®^ met all quality criteria for the expected value limits. Importantly, they observed that one of the ASNase products, that served as an example for the investigation of ASNase activity in a cohort of 62 patients, exhibited unsatisfactory therapeutic activity in vivo. This ASNase biogeneric also showed an estimated activity of the product that was 25% lower than specified in the product label [4]. We measured an enzyme activity of 70% for Onconase, which is a reduction in activity exceeding 25% compared to the claimed activity on the product label. Similar results were obtained by Sankaran et al. They showed that none of their tested generic asparaginase formulations constantly achieved the clinically defined threshold of 100 IU/L, and in vitro ASNase activity levels were as low as 71–74% [10, 15]. Thus, it can be assumed that Onconase is not suitable to act as a therapeutically effective treatment option in ALL, particularly in comparison with Spectrila^®^.

Another important finding in our analysis was that Spectrila^®^ showed extremely low levels of process-related impurities like *E*. *coli* host cell proteins, DNA and endotoxin far below the lower limits of the quality target values. This is essential because it is known that such impurities can be the cause of severe side effects due to their immunogenic potential. In fact, clinical hypersensitivity to *E*. *coli* ASNase treatment is one of the main reasons for therapy discontinuation due to swelling and redness at the injection site as well as anaphylaxis [16]. In addition, host cell proteins can act as an adjuvant to elicit a stronger anti-drug antibody response resulting in an increased risk of ASNase inactivation [11, 17]. In light of the allergic potential that *E*. *coli* ASNase formulations might exhibit, it is even more critical that ASNases show a very high purity and are free of any kind of contaminations.

Strikingly, Onconase exhibited impurity levels of host cell proteins, DNA and endotoxin levels, respectively, far above the accepted thresholds and is therefore not considered a safe pharmaceutical product to be used in ALL treatment.

To date, low- and middle-income countries depend on the availability of affordable ASNase formulations. Unfortunately, due to the favored use of comparably expensive pegylated ASNase in high-income countries, the supply of standard native *E*. *coli*-derived ASNase decreased worldwide [4]. As a consequence, ASNase formulations like Onconase, which raise concerns over their use in the treatment of pediatric ALL due to their poor-quality attributes, were brought to the markets in low- and middle-income countries. Our study showed that Onconase cannot be considered a comparable product to Spectrila^®^ from a quality point of view. To ensure safe clinical applicability, efficacy and safety of recombinant Spectrila^®^ was investigated in two clinical studies [18, 19]. Both studies compared Spectrila^®^ with a native ASNase (Asparaginase medac) and were able to demonstrate bioequivalence. In terms of safety, treatment with Spectrila^®^ did not lead to unexpected side effects. In fact, slightly fewer patients (-3.1% compared to Asparaginase medac) had ASNase-related adverse events [19]. For these reasons, Spectrila^®^ is a suitable and effective treatment in ALL. It remains questionable whether Onconase would fulfil the quality criteria for market authorization in Europe.

In conclusion, the head-to head investigation of Spectrila^®^ and the ASNase product Onconase revealed striking differences in their enzymatic activities, purity and safety. Spectrila^®^ was shown to be a recombinant *E*. *coli*-derived ASNase with high enzymatic activity, excellent purity and very low levels of process-related impurities. This investigation repeatedly demonstrated that the quality of Spectrila^®^ was unsurpassed in almost every aspect of the analysis and thus, Spectrila^®^ represents a safe treatment option in ALL.

## Supporting information

S1 Raw images (TIF)

## References

[1] Swain A, Jaskólski M, Housset D, Rao J, Wlodawer A. Crystal structure of Escherichia coli L-asparaginase, an enzyme used in cancer therapy. Proc Natl Acad Sci USA. 1993;90(4):1474–8. doi: 10.1073/pnas.90.4.1474 PubMed Central PMCID: PMC45896. 8434007 PMC45896

[2] Upadhyay AK, Singh A, Mukherjee KJ, Panda AK. Refolding and purification of recombinant L-asparaginase from inclusion bodies of E. coli into active tetrameric protein. Front Microbiol. 2014;5:486. Epub 2014/10/14. doi: 10.3389/fmicb.2014.00486 ; PubMed Central PMCID: PMC4164012. 25309524 PMC4164012

[3] Broome JD. Studies on the mechanism of tumor inhibition by L-asparaginase. Effects of the enzyme on asparagine levels in the blood, normal tissues, and 6C3HED lymphomas of mice: differences in asparagine formation and utilization in asparaginase-sensitive and -resistant lymphoma cells. J ExpMed. 1968;127(6):1055–72. doi: 10.1084/jem.127.6.1055 PubMed Central PMCID: PMC2138499. 4871211 PMC2138499

[4] Sidhu J, Gogoi MP, Agarwal P, Mukherjee T, Saha D, Bose P, et al. Unsatisfactory quality of E. coli asparaginase biogenerics in India: Implications for clinical outcomes in acute lymphoblastic leukaemia. Pediatr Blood Cancer. 2021;68(11):e29046. Epub 2021/05/04. doi: 10.1002/pbc.29046 ; PubMed Central PMCID: PMC7613163. 33939263 PMC7613163

[5] Egler RA, Ahuja SP, Matloub Y. L-asparaginase in the treatment of patients with acute lymphoblastic leukemia. J Pharmacol Pharmacother. 2016;7(2):62–71. Epub 2016/07/22. doi: 10.4103/0976-500X.184769 ; PubMed Central PMCID: PMC4936081. 27440950 PMC4936081

[6] EPAR. EMA/793954/2015, EMEA/H/C/002661.

[7] FDA. Pharmaceutical cGMPS for the 21st Century—A Risk-Based Approach. Rockville, MD. 2004b.

[8] FDA. Guidance for Industry PAT—A Framework for Innovative Pharmaceutical Development, Manufacturing, and Quality Assurance. Rockville, MD. 2004a.

[9] EMA. ICH guideline Q8 (R2) on pharmaceutical development. London. 2017.

[10] Sankaran H, Sengupta S, Purohit V, Kotagere A, Moulik NR, Prasad M, et al. A comparison of asparaginase activity in generic formulations of E.coli derived L- asparaginase: In-vitro study and retrospective analysis of asparaginase monitoring in pediatric patients with leukemia. Br J Clin Pharmacol. 2020;86(6):1081–8. Epub 2020/01/12. doi: 10.1111/bcp.14216 ; PubMed Central PMCID: PMC7256116. 31925802 PMC7256116

[11] Zenatti PP, Migita NA, Cury NM, Mendes-Silva RA, Gozzo FC, de Campos-Lima PO, et al. Low Bioavailability and High Immunogenicity of a New Brand of E. colil-Asparaginase with Active Host Contaminating Proteins. EBioMedicine. 2018;30:158–66. Epub 2018/03/20. doi: 10.1016/j.ebiom.2018.03.005 ; PubMed Central PMCID: PMC5952248. 29550241 PMC5952248

[12] Hustoft HK, Malerod H, Wilson SR, Reubsaet L, Lundanes E, Greibrokk T. A Critical Review of Trypsin Digestion for LC-MS Based Proteomics. In: Leung HCE, Man TK, Flores RJ, editors. Integrative Proteomics. Rijeka: IntechOpen; 2012. p. Ch. 4.

[13] Shifrin S, Parrott CL, Luborsky SW. Substrate binding and intersubunit interactions in L-asparaginase. J Biol Chem. 1974;249(5):1335–40. Epub 1974/03/10. . 4594124

[14] Yao H, Vandenbossche J, Sanger-van de Griend CE, Janssens Y, Fernandez CS, Xu X, et al. Development of a capillary zone electrophoresis method to quantify E. colil-asparaginase and its acidic variants. Talanta. 2018;182:83–91. Epub 2018/03/05. doi: 10.1016/j.talanta.2018.01.048 . 29501203

[15] van der Sluis IM, Vrooman LM, Pieters R, Baruchel A, Escherich G, Goulden N, et al. Consensus expert recommendations for identification and management of asparaginase hypersensitivity and silent inactivation. Haematologica. 2016;101(3):279–85. Epub 2016/03/02. doi: 10.3324/haematol.2015.137380 ; PubMed Central PMCID: PMC4815719. 26928249 PMC4815719

[16] Yen HJ, Chang WH, Liu HC, Yeh TC, Hung GY, Wu KH, et al. Outcomes Following Discontinuation of E. coli l-Asparaginase Upon Severe Allergic Reactions in Children With Acute Lymphoblastic Leukemia. Pediatr Blood Cancer. 2016;63(4):665–70. Epub 2015/12/26. doi: 10.1002/pbc.25869 . 26703788

[17] Bracewell DG, Francis R, Smales CM. The future of host cell protein (HCP) identification during process development and manufacturing linked to a risk-based management for their control. Biotechnol Bioeng. 2015;112(9):1727–37. Epub 2015/05/23. doi: 10.1002/bit.25628 ; PubMed Central PMCID: PMC4973824. 25998019 PMC4973824

[18] Pieters R, Appel I, Kuehnel HJ, Tetzlaff-Fohr I, Pichlmeier U, van der Vaart I, et al. Pharmacokinetics, pharmacodynamics, efficacy, and safety of a new recombinant asparaginase preparation in children with previously untreated acute lymphoblastic leukemia: a randomized phase 2 clinical trial. Blood. 2008;112(13):4832–8. Epub 2008/09/23. doi: 10.1182/blood-2008-04-149443 . 18805963

[19] van der Sluis IM, de Groot-Kruseman H, Te Loo M, Tissing WJE, van den Bos C, Kaspers GJL, et al. Efficacy and safety of recombinant E. coli asparaginase in children with previously untreated acute lymphoblastic leukemia: A randomized multicenter study of the Dutch Childhood Oncology Group. Pediatr Blood Cancer. 2018;65(8):e27083. Epub 2018/05/05. doi: 10.1002/pbc.27083 . 29727043

