# Supplementary figures and images for: Quality comparison of a state-of-the-art preparation of a recombinant L-asparaginase derived from Escherichia coli with an alternative asparaginase product
Source: PLoS One. 2023 Jun 15;18(6):e0285948. doi: 10.1371/journal.pone.0285948 (PMC10270636; doi:10.1371/journal.pone.0285948)

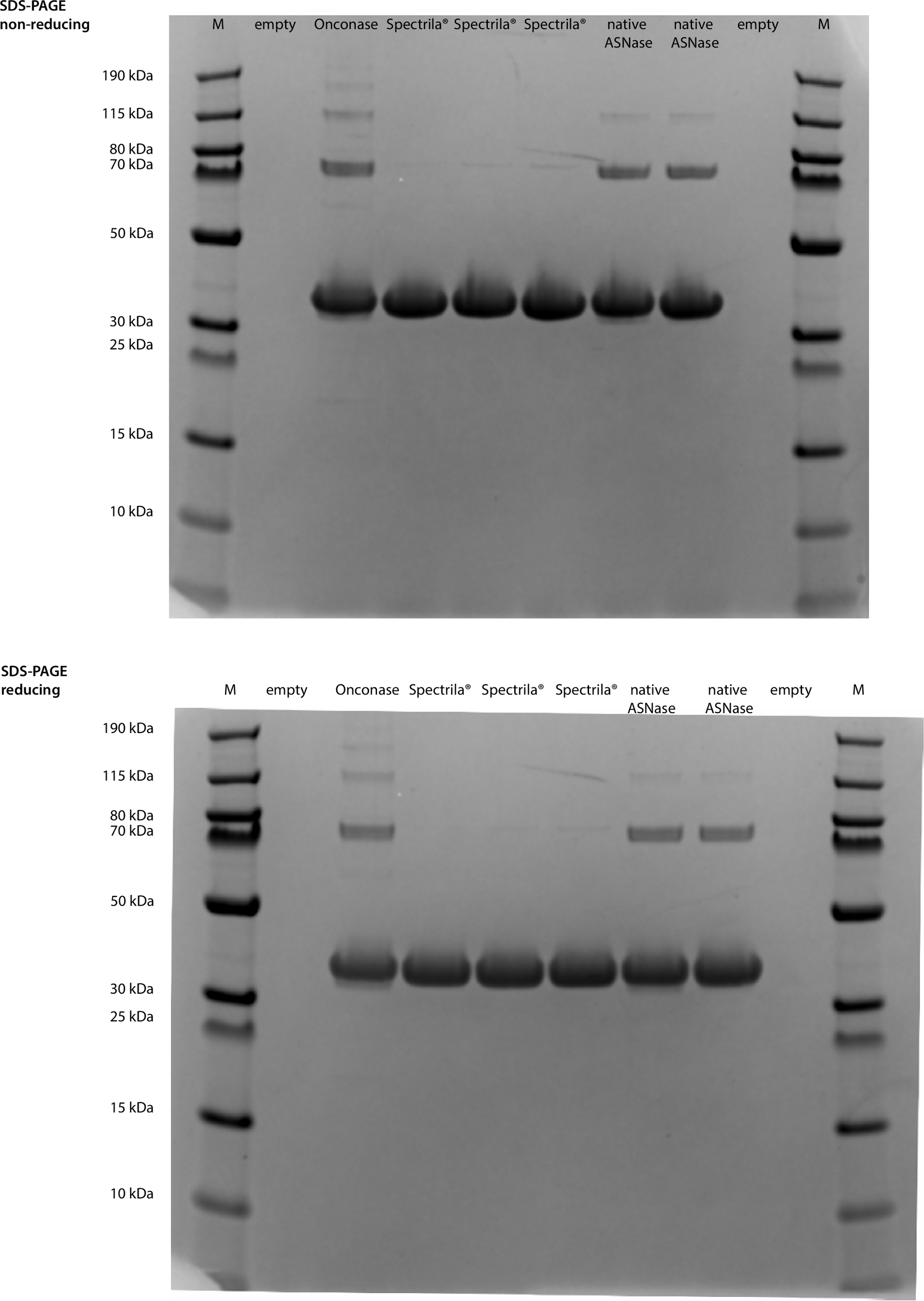

Supplement: S1 Raw images — (TIF) [file pone.0285948.s001.tif]
